# Diagnosis of Paralytic Rabies by Metagenomics Next‐Generation Sequencing: A Case Report and Review of the Literature

**DOI:** 10.1002/vms3.70748

**Published:** 2026-01-20

**Authors:** Lianghai Cao, Xingyu Wang, Youlian Zhou, Jun Qiu, Qianlv Zeng, Chaogui Zhang, Lingai Pan

**Affiliations:** ^1^ Department of Intensive Care Medicine Chengdu Integrated TCM & Western Medicine Hospital Chengdu Sichuan Province China; ^2^ Department of Intensive Care Medicine the Second People's Hospital of Yibin, West China Hospital Yibin China; ^3^ Department of Intensive Care Medicine Sichuan Provincial People's Hospital Chengdu China

**Keywords:** cerebrospinal fluid, mNGS, paralytic rabies, rabies virus

## Abstract

Paralytic rabies is an atypical form of the disease that is notoriously difficult to diagnose early due to the absence of classic features like hydrophobia. The case being discussed presents a patient who has altered mental status, for whom the initial diagnosis was difficult due to an absent clear bite history and typical symptoms. The final diagnosis of the case was confirmed by metagenomic next‐generation sequencing (mNGS) of directly from cerebrospinal fluid, which led to the detection of the rabies virus. This case underscores the critical diagnostic value of mNGS in identifying elusive neurotropic infections.

## Graphical abstract

This case report demonstrates the successful diagnosis of a challenging paralytic rabies case using metagenomic next‐generation sequencing (mNGS). mNGS provided a rapid and precise pathogen identification directly from cerebrospinal fluid where traditional methods failed. This highlights the critical role of mNGS as a powerful diagnostic tool for uncovering elusive neurotropic viruses and guiding clinical management in unexplained encephalitis cases.

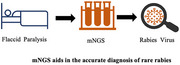

## 1 Introduction

Rabies is a neuroinfectious disease that causes approximately 60,000 human deaths per year (Chen et al. 2018; Jackson 2014). Domestic dogs remain the primary reservoir hosts for the virus across Eurasia and Africa, while vampire bats serve as the important reservoir in the Americas (Pfaff et al. 2019; Hemachudha et al. 2013; Wilde et al. 2013; Fooks et al. 2014). The disease manifests in two primary clinical forms: the encephalitic (‘furious’) type, which accounts for approximately 80% of cases, and the paralytic (‘dumb’) type, comprising the remaining 20% (Jackson 2014). The diagnosis of the paralytic Rabies form is particularly challenging for clinicians due to its absence of characteristic features, a difficulty compounded by the broad differential diagnosis of pathogens that can cause central nervous system infections (Dedkov et al. 2016; Wang et al. 2017). Metagenomic next‐generation sequencing (mNGS) has transformed the diagnosis of central nervous system (CNS) infections, enabling the detection of fastidious pathogens.

Despite its potential, the application of mNGS to frequently misdiagnosed diseases such as paralytic rabies is rarely documented. This case report aims to address this literature gap and highlight the utility of mNGS in diagnosing such challenging cases.

## 2 Case Presentation

One week previously, a 69‐year‐old female patient, who until then had been free from any significant health issues, was admitted to Community Hospital with complaints of numbness and dizziness, in addition to limb weakness. The patient reported no obvious cause for these symptoms. Four days prior, there was an exacerbation of the symptoms of numbness, accompanied by the onset of vomiting. One day before, the patient exhibited symptoms including visual hallucinations, incoherent speech, and a decline in consciousness (GCS 7/15: E2M4V1). On 10 October 2021, she was transferred to our emergency department. Upon admission, the patient's temperature was recorded at 36.5°C, while her pulse rate was documented as 88 beats per minute, her respiratory rate was 20 breaths per minute, and her blood pressure was 88/56 mmHg. A neurological examination was conducted, which revealed the following findings: the patient was comatose, with equally sized and round pupils (measuring diameter: 2.5 mm); the light reflexes were sluggish; and there was an absence of meningeal signs or focal deficits. The results of the laboratory examination revealed a white blood cell count (WBC) of 16.01 × 10⁹/L and a procalcitonin (PCT) concentration of 0.647 ng/mL. A blood gas analysis revealed the following concentrations: potassium ions (K+) at 3.4 mmol/L, sodium ions (Na+) at 130 mmol/L, and lactic acid (Lac) at 2.7 mmol/L. The liver function and renal function markers did not demonstrate any obvious abnormalities. The patient was found to have a negative antinuclear antibody titre. Head CT ruled out structural lesions, while chest CT demonstrated right lung infiltrates suggestive of pneumonia. Due to rapid neurological decline and suspected sepsis‐associated encephalopathy, she was transferred to the intensive care unit for further management. During the preliminary evaluation, the following neurological disorders were considered: intracranial infection and Guillain–Barré syndrome. Following admission, the patient was treated with protective tracheal intubation, mechanical ventilation, remifentanil analgesia, propofol sedation, norepinephrine to maintain blood pressure and ceftriaxone as an anti‐infective agent.

As of 11 October 2021, the patient's consciousness disorder remained unchanged. A lumbar puncture was conducted, yielding a clear and colourless cerebrospinal fluid (CSF). The CSF routine examination revealed a nucleated cell count of 28.00 × 10^6^/L, with a predominance of mononuclear cells (71%), and the CSF protein electrophoresis yielded a negative result. The biochemistry of the cerebrospinal fluid (CSF) revealed the following concentrations: lactate dehydrogenase, 41.0 U/L; glucose, 3.98 mmol/L (fingertip blood glucose, 6.5 mmol/L); adenosine deaminase (ADA), 1.9 U/L; chloride, 112.6 mmol/L; and total protein, 0.365 g/L. In light of the probable diagnosis of viral encephalitis, the therapeutic regimen was amended to include the addition of Ganciclovir, and cerebrospinal fluid (CSF) was dispatched to a third‐party testing company, namely the Sichuan Jinyu Medical Testing Center for unbiased mNGS Specifically, separate DNA and RNA libraries were sequenced on the Illumina NextSeq 550 platform. Pathogens were identified by aligning non‐human reads to a comprehensive database and were only reported if they met predefined criteria (≥3 unique reads, >1% genome coverage, and a significantly higher signal than negative controls).

**FIGURE 1:**
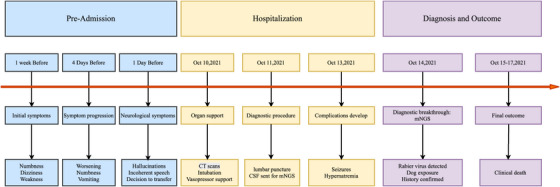
Patient treatment timeline.

On 13 October 2021, the patient exhibited signs of an epileptic seizure. In order to treat the condition, administration of the pharmaceutical drug Midazolam was initiated. Concurrently, the patient exhibited signs of hypernatraemia, necessitating the administration of sodium‐lowering agents to address the condition. On 14 October 2021, the mNGS assay detected nucleic acids of Rabies lyssavirus in the CSF, as evidenced by 11 unique reads and a genome coverage of 1.31% (156/11926). Sequences aligning to Epstein–Barr virus (EBV) were also identified (12 reads; 0.4289% coverage; 92.31% relative abundance), the clinical significance of which remains unclear (see Table S1 for details). A detailed medical history was obtained from the patient's family members. The patient exhibited a documented history of exposure to dog scratches and bites, with the most recent occurrence occurring over one year ago, and the previous one occurring over a decade ago. The patient did not receive the vaccine for rabies in a timely fashion. The final diagnosis was paralytic rabies. On 15 October 2021, the patient's consciousness deteriorated to a profound coma, with a GCS score of 3 points. The pupils were found to be bilaterally equal in size and shape, exhibiting an approximate diameter of 4 millimetres. The presence of the pupillary light reflex was not observed. On the 7th day after the patient's admission to the hospital, she succumbed to central respiratory and circulatory failure. The temporal progression of the disease is illustrated in Figure 1.

## 3 Discussion

The clinical stages of rabies are as follows: incubation period, prophase of clinical symptoms (prodrome), acute nervous period, coma period, and death (Hemachudha et al. 2013). The symptoms of furious rabies are characterised by changes in consciousness and mental state, hydrophobia, acrophobia, and symptoms of autonomic nerve stimulation, and the most specific feature is hydrophobia (Jackson 2014). Paralytic rabies, on the other hand, is distinguished by its absence of the aforementioned characteristics and its clinical manifestations are mimicking Guillain‐Barré syndrome (Pannu et al. 2019; Kumar et al. 2019, Mahadevan et al. 2016).

The primary diagnostic method for rabies is the use of fluorescent antibody staining to detect rabies virus antigen in skin biopsy samples. Furthermore, the presence of neutralising anti‐rabies virus antibodies in the cerebrospinal fluid (CSF) is widely regarded as substantial evidence of rabies encephalitis. Additionally, the detection of rabies virus RNA in saliva by reverse transcription polymerase chain reaction (RT‐PCR) is a significant diagnostic test (Jackson 2014). However, it should be noted that the aforementioned laboratory methods are frequently only able to detect the disease in its late stages, and a negative test result cannot exclude the diagnosis of rabies. Metagenomic next‐generation sequencing (mNGS), also termed clinical metagenomics, is characterised by the parallel sequencing of all nucleic acids (DNA and/or RNA) within a specimen. Subsequently, the sequenced data are compared against known microbial databases, allowing for the identification of microorganisms present in the sample and enabling the unbiased detection of pathogens (Simner et al. 2018; Zinter et al. 2019). As a recently developed technology, mNGS has been employed in clinical practice in recent years. The present methodology has been demonstrated to exceed current microbial diagnostic methods in identifying pathogens that are recalcitrant to cultivation and conducive to the expeditious identification of pathogens (Xu et al. 2020; Yi et al. 2020).

Furthermore, in this study, Epstein–Barr virus (EBV) was incidentally detected in the patient's cerebrospinal fluid (CSF) by mNGS. Although EBV is recognised as a causative agent for certain central nervous system diseases, its detection in the CSF must be interpreted with caution. It is important to note that the presence of EBV‐DNA does not necessarily indicate causation, especially in the absence of serological evidence of primary infection or a positive in situ hybridisation result for Epstein–Barr virus‐encoded small RNAs (EBERs) in pathological tissue (Wang et al. 2022; Chinese Society of Medical Virology, Chinese Society of Infectious Diseases, Chinese Medical Association 2018). Furthermore, the prevalence of EBV infection is over 90% in the adult population worldwide, while its direct involvement in nervous system disease is uncommon and occurs predominantly in paediatric populations (Nakamura et al. 2017; Baldwin and Cummings 2018; Luzuriaga and Sullivan 2010). Consequently, its presence in the CSF may frequently be attributable to nonspecific viral reactivation, particularly in critically ill patients, those with immune dysfunction, or in the context of another primary central nervous system insult. In this context, the detection of EBV is more likely to reflect immunosuppression resulting from the patient's severe systemic or central nervous system disease state than to represent a primary etiology(Soldan and Lieberman 2023). Therefore, on the basis of the epidemiological and clinical context, Epstein–Barr virus (EBV) is considered an unlikely causative agent in this case, and the primary etiology is confidently attributed to the rabies virus.

A comprehensive review of the literature on rabies cases was conducted, which resulted in the identification of eight cases, as outlined in Table S2 (Chen et al. 2018; Dedkov et al. 2016; Regnault et al. 2022; McDermid et al. 2008; Pin et al. 2022; Tricou et al. 2014). Of the eight subjects, six were male and two were female, with ages ranging from 7 to 73 years. With regard to exposure, three cases had a documented history of animal bites, one case involved bat exposure, two transplant recipients had donors with a history of animal bites, and two did not report any such history. Five patients presented with furious rabies, and three presented with paralytic rabies. Of the eight patients, seven were diagnosed using PCR and seven by NGS, with six receiving both tests. It is worthy of note that one patient was initially found to be negative on the initial mNGS test upon admission, yet subsequently returned a positive result on the saliva‐based mNGS test performed on the fifth day. Furthermore, three patients succumbed to encephalitis of an unknown etiology, with the diagnosis only being confirmed post‐mortem. With the exception of one patient who was not available for further follow‐up, all seven remaining patients died.

These fatal rabies cases reveal critical gaps in prevention. Infections following known exposures indicate failures in timely prophylaxis, whereas cases without clear bites, including those of a transplanted origin, demonstrate variable transmission routes. The post‐mortem diagnosis of three patients with unexplained encephalitis highlights the profound diagnostic challenge, which is further compounded by the limitations of initial testing, as evidenced by delayed positive results. Consequently, the utilisation of repeated or multimodal molecular testing, such as broad‐range PCR and NGS, is imperative for clinically suspected cases. Collectively, these findings not only emphasise the urgent need for enhanced public awareness and vigilance among healthcare professionals to prevent fatalities but also offer new insights for clinicians managing rare diseases.

## Author Contributions

LC and LP were involved in the conception and design of the work. XW wrote the first draft of the manuscript and revised it. LC and LP revised the manuscript. QZ and CZ were responsible for the management of this patient. YZ, JQ, and QZ collected the clinical data. LH and XW reviewed the literature throughout this study. All authors contributed to the article and approved the submitted version.

## Funding

The authors have nothing to report.

## Conflicts of Interest

The authors declare that the research was conducted in the absence of any commercial or financial relationships that could be construed as a potential conflict of interest.

## Supporting information




**Supporting Table 1**: Detection report of pathogenic microorganisms by mNGS.
**Supporting Table 2**: Summary of case reports detecting rabies virus via mNGS in PubMed literature.

## Data Availability

The original contributions presented in the study are included in the article. Further inquiries can be directed to the corresponding author.

## References

[vms370748-bib-0001] Baldwin, K. J. , and C. L. Cummings . 2018. “Herpesvirus Infections of the Nervous System.” Continuum (Minneap Minn) 24: 1349–1369. 10.1212/CON.0000000000000661. 30273243

[vms370748-bib-0002] Chen, J. , G. Liu , and T. Jin , et al. 2018. “Epidemiological and Genetic Characteristics of Rabies Virus Transmitted Through Organ Transplantation.” Frontiers in Cellular and Infection Microbiology 8: 86. 10.3389/fcimb.2018.00086. 29637047 PMC5880885

[vms370748-bib-0003] Chinese Society of Medical Virology, Chinese Society of Infectious Diseases, Chinese Medical Association . 2018. “Expert Consensus on Laboratory Diagnosis and Clinical Application of Epstein‐Barr Virus Infection.” Chinese Journal of Experimental and Clinical Virology 32, no. 1: 2–8.

[vms370748-bib-0004] Dedkov, V. G. , A. N. Lukashev , A. A. Deviatkin , et al. 2016. “Retrospective Diagnosis of Two Rabies Cases in Humans by High Throughput Sequencing.” Journal of Clinical Virology 78. 10.1016/j.jcv.2016.03.012. 26998568

[vms370748-bib-0005] Fooks, A. R. , A. C. Banyard , D. L. Horton , N. Johnson , L. M. McElhinney , and A. C. Jackson . 2014. “Current Status of Rabies and Prospects for Elimination.” Lancet 384, no. 9951: 1389–1399. 10.1016/S0140-6736(13)62707-5. 24828901 PMC7159301

[vms370748-bib-0006] Hemachudha, T. , G. Ugolini , S. Wacharapluesadee , W. Sungkarat , S. Shuangshoti , and J. Laothamatas . 2013. “Human Rabies: Neuropathogenesis, Diagnosis, and Management.” Lancet Neurology 12, no. 5: 498–513. 10.1016/S1474-4422(13)70038-3. 23602163

[vms370748-bib-0007] Jackson, A. C. 2014. “Rabies.” Handbook of Clinical Neurology 123: 601–618. 10.1016/B978-0-444-53488-0.00029-8. 25015507

[vms370748-bib-0008] Kumar, N. , P. Gupta , and M. K. Meena . 2019. “Paralytic Rabies: A Guillain‐Barre Syndrome Mimic.” Qjm 112, no. 5: 365–366. 10.1093/qjmed/hcz054. 30830154

[vms370748-bib-0009] Luzuriaga, K. , and J. L. Sullivan . 2010. “Infectious Mononucleosis.” New England Journal of Medicine 362, no. 21: 1993–2000. 10.1056/NEJMcp1001116. 20505178

[vms370748-bib-0010] Mahadevan, A. , M. S. Suja , R. S. Mani , and S. K. Shankar . 2016. “Perspectives in Diagnosis and Treatment of Rabies Viral Encephalitis: Insights From Pathogenesis.” Neurotherapeutics 13, no. 3: 477–492. 10.1007/s13311-016-0452-4. 27324391 PMC4965414

[vms370748-bib-0011] McDermid, R. C. , L. Saxinger , B. Lee , et al. 2008. “Human Rabies Encephalitis Following Bat Exposure: Failure of Therapeutic Coma.” Cmaj 178, no. 5: 557–561. 10.1503/cmaj.071326. 18299541 PMC2244651

[vms370748-bib-0012] Nakamura, Y. , H. Nakajima , H. Tani , et al. 2017. “Anti‐MOG Antibody‐Positive ADEM Following Infectious Mononucleosis due to a Primary EBV Infection: A Case Report.” BMC Neurology [Electronic Resource] 17, no. 1: 76. 10.1186/s12883-017-0858-6. 28420330 PMC5395865

[vms370748-bib-0013] Pannu, A. K. , R. V. Kumar , D. Vijaykumar , L. Priya , H. Singh , and A. Bhalla . 2019. “Paralytic Rabies: An Acute Flaccid Myelitis After Inadequate Post Exposure Prophylaxis.” Tropical Doctor 49, no. 4: 301–302. 10.1177/0049475519852223. 31132966

[vms370748-bib-0014] Pfaff, F. , T. Müller , C. M. Freuling , et al. 2019. “In‐Depth Genome Analyses of Viruses From Vaccine‐Derived Rabies Cases and Corresponding Live‐Attenuated Oral Rabies Vaccines.” Vaccine 37, no. 33: 4758–4765. 10.1016/j.vaccine.2018.01.083. 29439868

[vms370748-bib-0015] Pin, L. , X. Lutao , L. Linjie , P. Qunjie , F. Weijun , and D. Wang . 2022. “A New Choice for human Rabies Diagnosis: A Case Report of Metagenomics Next‐Generation Sequencing in Diagnosis of human Rabies.” Journal of Infection and Public Health 15, no. 11: 1276–1278. 10.1016/j.jiph.2022.10.003. 36272393

[vms370748-bib-0016] Regnault, B. , B. Evrard , I. Plu , et al. 2022. “First Case of Lethal Encephalitis in Western Europe due to European Bat Lyssavirus Type 1.” Clinical Infectious Diseases 74, no. 3: 461–466. 10.1093/cid/ciab443. 33991184

[vms370748-bib-0017] Simner, P. J. , S. Miller , and K. C. Carroll . 2018. “Understanding the Promises and Hurdles of Metagenomic Next‐Generation Sequencing as a Diagnostic Tool for Infectious Diseases.” Clinical Infectious Diseases 66, no. 5: 778–788. 10.1093/cid/cix881. 29040428 PMC7108102

[vms370748-bib-0018] Soldan, S. S. , and P. M. Lieberman . 2023. “Epstein‐Barr Virus and Multiple Sclerosis.” Nature Reviews Microbiology 21, no. 1: 51–64. 10.1038/s41579-022-00770-5. 35931816 PMC9362539

[vms370748-bib-0019] Tricou, V. , N. Berthet , E. Nakouné , and M. Kazanji . 2014. “Complete Genome Sequence of a Rabies Virus Isolated From a Human in Central African Republic.” Genome announcements 2, no. 3: e00598–14. 10.1128/genomeA.00598-14. PMC4064799 24948767

[vms370748-bib-0020] Wang, J. , P. Yu , Z. Xie , et al. 2017. “A Resequencing Pathogen Microarray Method for High‐Throughput Molecular Diagnosis of Multiple Etiologies Associated With Central Nervous System Infection.” Archives of Virology 162, no. 12: 3769–3778. 10.1007/s00705-017-3550-7. 28913577 PMC7087039

[vms370748-bib-0021] Wang, Y. , J. Yang , and Y. Wen . 2022. “Lessons From Epstein‐Barr Virus DNA Detection in Cerebrospinal Fluid as a Diagnostic Tool for EBV‐Induced Central Nervous System Dysfunction Among HIV‐Positive Patients.” Biomedicine & Pharmacotherapy 145: 112392. 10.1016/j.biopha.2021.112392. 34781140

[vms370748-bib-0022] Wilde, H. , T. Hemachudha , S. Wacharapluesadee , B. Lumlertdacha , and V. Tepsumethanon . 2013. “Rabies in Asia: The Classical Zoonosis.” Current Topics in Microbiology and Immunology 365: 185–203. 10.1007/82_2012_228. 22678037

[vms370748-bib-0023] Xu, A. , H. Zhu , B. Gao , et al. 2020. “Diagnosis of Severe Community‐Acquired Pneumonia Caused by *Acinetobacter baumannii* Through next‐Generation Sequencing: A Case Report.” BMC Infectious Diseases [Electronic Resource] 20, no. 1: 45. 10.1186/s12879-019-4733-5. 31941459 PMC6964051

[vms370748-bib-0024] Yi, H. , J. Fang , J. Huang , B. Liu , J. Qu , and M. Zhou . 2020. “Legionella Pneumophila as Cause of Severe Community‐Acquired Pneumonia, China.” Emerging Infectious Diseases 26, no. 1: 160–162. 10.3201/eid2601.190655. 31855541 PMC6924908

[vms370748-bib-0025] Zinter, M. S. , C. C. Dvorak , M. Y. Mayday , K. Iwanaga , N. P. Ly , and M. E. McGarry . 2019. “Pulmonary Metagenomic Sequencing Suggests Missed Infections in Immunocompromised Children.” Clinical Infectious Diseases 68, no. 11: 1847–1855. 10.1093/cid/ciy802. 30239621 PMC6784263

