# Supplementary material for: Diagnosis of Paralytic Rabies by Metagenomics Next‐Generation Sequencing: A Case Report and Review of the Literature
Source: Vet Med Sci. 2026 Jan 20;12(1):e70748. doi: 10.1002/vms3.70748 (PMC12817918; doi:10.1002/vms3.70748)
Supplement: Supplementary file 1 — Supporting Table 1: Detection report of pathogenic microorganisms by mNGS. Supporting Table 2: Summary of case reports detecting rabies virus via mNGS in PubMed literature. [file VMS3-12-e70748-s001.docx]

Supplementary Table1. Detection report of pathogenic microorganisms by mNGS

| Detection results | | | | | | |
| --- | --- | --- | --- | --- | --- | --- |
| 1. List of virus DNA detected | | | | | | |
| Genus | | | Species | | | |
| Latin name | Reads | Relative abundance | Latin name | Reads | Genome coverage |  |
| Lympho- cryptovirus | 12 | 92.31% | Human gamma herpesvirus 4 (EBV) | 12 | 0.4289%(741/172764) |  |
| 2. List of virus RNA detected | | | | | | |
| Genus | | | Species | | | |
| Latin name | Reads | Relative abundance | Latin name | Reads | Genome coverage |  |
| Lyssa virus | 11 | 9.57% | Rabies lyssa virus | 11 | 1.3081%(156/11926) |  |

Supplementary Table 2: Summary of Case Reports Detecting Rabies Virus via mNGS in PubMed Literature

| ^References^ | ^Age (years)^ | ^Gender^ | ^Infection mode^ | ^Incubation period^ | ^Clinical manifestation^ | ^Time from onset to diagnosis (days)^ | ^Sample type^ | ^Routine methods of rabies diagnosis^ | ^Reads^ | ^Outcome^ |
| --- | --- | --- | --- | --- | --- | --- | --- | --- | --- | --- |
| ^McDermid et al., 2008.^ | ^73^ | ^M^ | ^Bat^ | ^6 months^ | ^Dysphagia^ | ^12^ | ^Neck skin, saliva and serum samples^ | ^PCR^ | ^Not^  ^mentioned^ | ^Death^ |
| ^Tricou et al., 2014^ | ^Not^  ^mentioned^ | ^M^ | ^Dog^ | ^A few weeks^ | ^Not classified, only described as mental confusion^ | ^Not^  ^mentioned^ | ^Brain tissue^ | ^PCR+NGS^ | ^Not^  ^mentioned^ | ^Not mentioned^ |
| ^Dedkov et al., 2016^ | ^7^ | ^M^ | ^Not^  ^mentioned^ | ^Not^  ^mentioned^ | ^Not described, diagnosed as unexplained encephalitis^ | ^Not^  ^mentioned^ | ^Brain tissue^ | ^PCR^ | ^124,781^ | ^Death^ |
| ^Dedkov et al., 2016^ | ^50^ | ^M^ | ^Not^  ^mentioned^ | ^Not^  ^mentioned^ | ^Not described, diagnosed as unexplained encephalitis^ | ^Not^  ^mentioned^ | ^Brain tissue^ | ^PCR^ | ^124,781^ | ^Death^ |
| ^Chen et al.,2018^ | ^47^ | ^F^ | ^Dog bite donor transmitted to renal transplant recipient^ | ^44 days^ | Hypodynamia | ^2^ | ^Saliva sample^ | ^PCR+NGS^ | ^772^ | ^Death^ |
| ^Chen et al., 2018^ | ^29^ | ^F^ | ^Dog bite donor transmitted to renal transplant recipient^ | ^44 days^ | ^Vomiting^ | ^5^ | ^Saliva sample^ | ^PCR+NGS^ | ^37^ | ^Death^ |
| ^Regnault et al., 2021^ | ^59^ | ^M^ | ^Bat^ | ^Not^  ^mentioned^ | ^Asthenia^ | ^Not mentioned^ | ^Brain tissue^ | ^NGS^ | ^Not^  ^mentioned^ | ^Death^ |
| ^Pin et al., 2022^ | ^59^ | ^M^ | ^Dog^ | ^20 days^ | ^Aggression^ | ^5^ | ^Neck skin, cerebrospinal fluid, Saliva sample^ | ^NGS^ | ^Not^  ^mentioned^ | ^Death^ |
